# Diverging effects of premature birth and bronchopulmonary dysplasia on exercise capacity and physical activity – a case control study

**DOI:** 10.1186/s12931-019-1238-0

**Published:** 2019-11-21

**Authors:** Katharina Ruf, Wolfgang Thomas, Maximilian Brunner, Christian P. Speer, Helge Hebestreit

**Affiliations:** 1 University Children’s Hospital Würzburg, University of Würzburg, Josef-Schneider-Str. 2, 97080 Würzburg, Germany; 2 Abteilung für Kinder- und Jugendmedizin, Klinikum Mutterhaus der Borromäerinnen, Feldstr. 16, 54290 Trier, Germany; 3 Klinik für Allgemein- und Viszeralchirurgie, Universitätsklinikum Erlangen, Krankenhausstraße 12, 91054 Erlangen, Germany

**Keywords:** Bronchopulmonary dysplasia, Physical activity, Exercise testing, Preterm birth, Exercise capacity, Sedentary behaviour

## Abstract

**Background:**

Extreme prematurity has been associated with exercise intolerance and reduced physical activity. We hypothesized that children with bronchopulmonary dysplasia (BPD) would be especially affected based on long-term lung function impairments. Therefore, the objective of this study was to compare exercise capacity and habitual physical activity between children born very and extremely preterm with and without BPD and term-born children.

**Methods:**

Twenty-two school-aged children (aged 8 to 12 years) born with a gestational age < 32 weeks and a birthweight < 1500 g (9 with moderate or severe BPD (=BPD), 13 without BPD (=No-BPD)) and 15 healthy term-born children (=CONTROL) were included in the study. Physical activity was measured by accelerometry, lung function by spirometry and exercise capacity by an incremental cardiopulmonary exercise test.

**Results:**

Peak oxygen uptake was reduced in the BPD-group (83 ± 11%predicted) compared to the No-BPD group (91 ± 8%predicted) and the CONTROL group (94 ± 9%predicted). In a general linear model, variance of peak oxygen uptake was significantly explained by BPD status and height but not by prematurity (*p* < 0.001).

Compared to CONTROL, all children born preterm spent significantly more time in sedentary behaviour (BPD 478 ± 50 min, No-BPD 450 ± 52 min, CONTROL 398 ± 56 min, *p* < 0.05) and less time in moderate-to-vigorous-physical activity (BPD 13 ± 8 min, No-BPD 16 ± 8 min, CONTROL 33 ± 16 min, *p* < 0.001). Prematurity but not BPD contributed significantly to explained variance in a general linear model of sedentary behaviour and likewise moderate-to-vigorous-physical activity (*p* < 0.05 and *p* < 0.001 respectively).

**Conclusion:**

In our cohort, BPD but not prematurity was associated with a reduced exercise capacity at school-age. However, prematurity regardless of BPD was related to less engagement in physical activity and more time spent in sedentary behaviour. Thus, our findings suggest diverging effects of prematurity and BPD on exercise capacity and physical activity.

## Background

Preterm birth has been associated with long-term sequelae such as pulmonary function impairment due to bronchopulmonary dysplasia (BPD) [1]. However, effects of prematurity on other outcomes such as exercise capacity are less clear and studies have provided conflicting results [2–8]. An important limitation of most studies was that children were included regardless of lung disease severity as BPD status and severity was rarely reported. Furthermore, the picture of BPD has changed over time.

The “classical” BPD described by Northway in 1967 was characterized by a diffuse and severe damage to the lung leading to focal hyperinflation and interstitial fibrosis [9]. With the introduction of antenatal steroids for lung maturation and surfactant therapy for respiratory distress syndrome in the 1990s, the pattern of defects has changed. This “new” BPD is characterised by changes in pulmonary vascularization and alveolarization based on an arrest of lung development [10, 11], which lead to impaired gas exchange and a possible need for supplemental oxygen in early infancy [1, 12]. The new BPD is due to lower gestational age and lower birth weight together with pre- and postnatal factors such as chorioamnionitis, sepsis, mechanical ventilation, oxygen therapy and inflammation [12–14].

During childhood, BPD has been associated with bronchial obstruction and reduced diffusion capacity [3, 5, 12, 15]. Lung function impairment may persist into adult age [16, 17]. Thus, BPD affects health beyond the neonatal period and is relevant for long term outcome after extreme prematurity [16]. Given persisting impairments in lung function and alveolar vascularization in children with BPD born in the late 1990s and thereafter, a reduced exercise capacity can be hypothesized.

Similar to exercise capacity, existing data on physical activity in former preterm children show diverging results. Reduced physical activity has been reported in teens and adults who were born preterm at very low birthweight [18, 19], possibly related to constraints such as neuromotor, cognitive or airway abnormalities. Other studies, however, report no differences in objectively measured physical activity when comparing school-age children born preterm to term-born children [3, 20]. The effects of BPD on physical activity have not been assessed yet. However, given the more pronounced and persistent pulmonary impairments in children born prematurely with BPD compared to those without BPD, we hypothesised limitations in physical activity especially in children with BPD.

To test our above hypotheses, this study assessed exercise capacity and objectively measured physical activity in children born extremely preterm with moderate or severe BPD and without BPD in comparison to term-born children.

## Materials and methods

### Study population

**Fig. 1:**
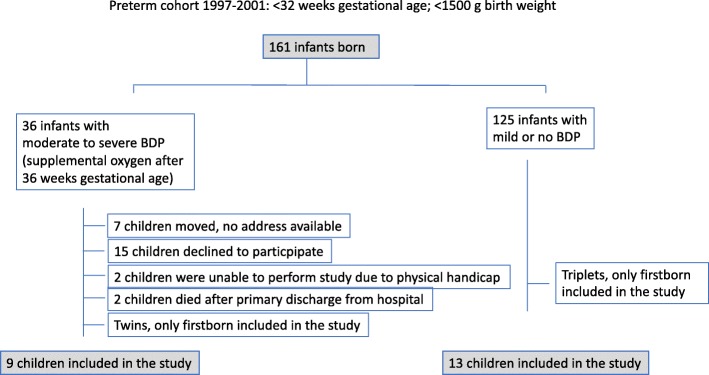
Recruitment of participants

All surviving preterm infants with a gestational age < 32 completed weeks and a birth weight < 1500 g treated in our hospital in the years 1997 to 2001 were eligible for the study. 46 of these met the criteria of moderate or severe BPD as defined by Jobe and Bancalari [1]. For recruitment, a letter of invitation was sent to the latest available address. Finally, 10 children with moderate to severe BPD (BPD) and their legal guardians consented to participate. Of the same premature cohort, 15 children without BPD (No-BPD) were included in the study. Since twins were initially included in the BPD group and triplets in the No-BPD group and – according to the analysis plan – only the first-borns were chosen for analysis, 3 children were excluded. Recruitment details are shown in Fig. 1. Furthermore, 15 healthy children born at term between 1997 and 2001 with an uneventful neonatal period served as controls (CONTROL). These were friends or siblings of the included preterm children to reduce selection bias. At the time of assessment, participants were aged 7–12 years.

### Study design

The study was approved by the Ethics Committee of the Medical Faculty of the University of Würzburg (46/09). Verbal assent and written informed consent were obtained from participants and their legal guardians, respectively.

Participants came for one study visit to our hospital. After physical examination and taking anthropometric measures, the children completed lung function testing including spirometry, body plethysmography and assessment of diffusion capacity. Subsequently, after familiarizing the participants with the equipment, they performed an incremental exercise test on a cycle ergometer up to volitional fatigue.

Following the study visit, physical activity was continuously measured by accelerometry, which allowed objectively recording activity in a free-living environment.

### Assessment of physical activity by accelerometry

Accelerometry took place for all participants in October and November 2009 to avoid bias due to seasonal variations of physical activity [21]. Study participants wore ActiGraph GT1M accelerometers (ActiGraph, Fort Walton Beach, Florida, USA). They were instructed to wear the device on the right hip for 9 consecutive days during waking hours and with the exception of water-related activities. Activity counts were averaged over a period of 15 s (epochs) and counts/minute were calculated from these data. A minimum recording time of 10 h on at least 3 week-days and 1 weekend-day was considered to yield valid data. Sedentary activity was defined as ≤100cts/min, light activity as > 100 ≤ 1000 cts/min. Time spent in moderate and vigorous physical activity (MVPA) was derived with the lower threshold for MVPA set at 3600 cpm (equal to 4 metabolic equivalents of task (METs)), according to a calibration study with Caucasian children of similar age [22].

### Anthropometry

Height was measured using a fixed stadiometer (Seca216, Seca, Hamburg, Germany). Weight was recorded in underwear to the nearest 0.1 kg using a digital scale (Seca701, Seca, Hamburg, Germany). Skinfold measurements were performed in triplicates at clearly defined sites (suprailiac, subscapular, over the biceps and over the triceps; Tanner/Whitehouse skinfold caliper, Holtain, Crymych, UK) and the median was used to estimate the children’s fat free body mass [23].

### Physical examination, electrocardiography and echocardiography

A complete medical history was taken including information on physician-diagnosed asthma, severe visual or hearing impairments or cerebral palsy. A thorough physical examination was conducted and a 12-lead electrocardiography (Custocard M, Customed, Ottobrunn, Germany) and echocardiography (VIVID 7-System, General Electric Healthcare, Milwaukee/WI, USA) were performed to identify contraindications against maximal exercise testing.

### Lung function testing

The Jaeger MasterScreen System (CareFusion, Hoechberg, Germany) was used to measure spirometric data, static lung volumes, airway resistance and diffusing capacity for carbon monoxide (TLCO). Spirometry has been applied to assess forced expiratory volume in 1 s (FEV1) and forced vital capacity (FVC), which reflect a possible obstructive or restrictive airway disease. Likewise, static lung volumes were assessed using bodyplethysmography. Residual volume (RV) and total lung capacity (TLC) were analysed to gain information about restrictive airway disease or a possible hyperinflation. Especially, RV to TLC ratio (RV%TLC) indicates a pulmonary hyperinflation. Specific airway resistance (SReff) has served as further marker of airway obstruction. Diffusion capacity (TLCOC) has been measured with the help of the diffusion capacity for carbon monoxide in the single breath technique (10 s breath hold). All lung function testing was performed according to current standards [24], values are expressed as percent of predicted values [25, 26], limits of normal ranges were determined according to Pellegrino et al. [27].

Study participants did not use regular asthma medications and none of them used inhaled bronchodilators prior to lung function testing. As our study protocol precluded blood drawing, TLCO was not corrected for haemoglobin-concentrations. Further, exhaled nitric oxide (eNO) as a marker of eosinic inflammation was measured (Eco medical, Analyzer CLD 88 sp., Duernten, Switzerland).

### Exercise testing

For cycle ergometry, a calibrated cycle ergometer (Ergoselect 200OK, Ergoline, Bitz, Germany) was used with the seat adjusted in horizontal and vertical position and the crank arm length modified so that the legs were bent not more than 90° and not fully extended during cycling. All participants completed a previously published continuous incremental exercise test protocol [28]. The initial work rate was 7 W for 2 min. It was increased to 1 W∙kg^− 1^ body weight for 2 min, 2 W∙kg^− 1^ body weight for another 2 min and then by 0.5 W∙kg^− 1^ body weight every minute up to volitional fatigue. A maximal effort was assumed if the subjects’ appearance suggested maximal exertion and their heart rate was above 195/min and/or their respiratory exchange rate (RER) exceeded 1.03 [29].

Ventilatory and gas exchange parameters were measured breath-by-breath using a metabolic cart (CPX/D, MedGraphics). VO_2_peak was taken as the highest VO_2_ over 30 s during the exercise test and expressed as %predicted based on gender and height [29]. The workload (in Watt) of the last completed stage of the incremental test was equally transformed into %predicted (maximal workload) [30].

### Statistical analysis

Participants’ characteristics are described by means and standard deviation as data was normally distributed. To assess differences in continuous variables among the three groups, ANOVA analyses were calculated. Posthoc t-tests with Bonferroni adjustments of *p*-values served to detect differences between the groups BPD, No-BPD, and CONTROL.

To assess influences of BPD and premature birth on exercise capacity and physical activity, general linear models were used. Peak oxygen uptake was set as dependent variables, height, premature birth (yes/no) and status of bronchopulmonary dysplasia (yes/no) were entered as independent variables. Likewise, time spent in sedentary behaviour or MVPA, respectively, were used as dependent variables with height, premature birth, and status of bronchopulmonary dysplasia as independent variables. Further, we tested body mass index (BMI) instead of height in a linear model as a possible marker for obesity when analysing sedentary behaviour. Effects of the respective variables on the model fit are reported as partial eta squared.

All statistical analyses were performed using SPSS version 25 (SPSS Inc., Chicago, USA). A *p*-value < 0.05 was considered significant.

## Results

The participants’ characteristics are presented in Table 1. No differences were found among BPD, No-BPD and CONTROL with regard to age, sex, heart rate at rest, blood pressure, height, weight or fat free body mass.

**Table 1:** Patients’ characteristics

	BPD *n* = 9	No-BPD *n* = 13	CONTROL *n* = 15	*p*-value
Age (years)	10.9 ± 1.7	10.4 ± 1.5	9.9 ± 1.3	0.22
Male (n (percentage of total))	4 (40%)	7 (47%)	8 (53%)	0.85
Heart rate (beats∙min^− 1^)	83.9 ± 8.4	76.5 ± 13.5	82.7 ± 14.7	0.29
Mean blood pressure (mmHg)	85.0 ± 8.0	84.2 ± 11.4	82.5 ± 4.0	0.74
Height (cm)	140.2 ± 11.0	144.6 ± 10.8	140.2 ± 9.5	0.26
Weight (kg)	32.6 ± 11.9	34.4 ± 7.4	31.7 ± 6.6	0.20
Fat free body mass (kg)	25.4 ± 5.8	28.0 ± 5.6	26.4 ± 5.2	0.50

Data are shown as number (n) and percentage of total or mean ± standard deviation

Data on the ante-and perinatal period are displayed in Table 2.

**Table 2:** pre-and perinatal data

	BPD *n* = 9	No-BPD *n* = 13	CONTROL *n* = 15
Gestational age (weeks)	26.6 ± 1.6 (24.6–29.7)	29.1 ± 1.8 (25.0–31.0)	39.3 ± 0.9 (38.0–41.0)
Birthweight (g)	766.7 ± 212.4 (450–1080)	117.3 ± 242.6 (780–1440)	3382.7 ± 327.5 (2800–3950)
SGA (number of total/percent)	3/9 (33%)	3/13 (23%)	3/15 (20%)
Antenatal steroids (number of total/percent)	5/9 (56%)	7/13 (54%)	0
Surfactant (number of total/percent)	8/9 (89%)	4/13 (31%)	0
Respiratory support			
Synchronized-intermittent-mandatory ventilation (SIMV) Number of patients/days	9/9 24.9 ± 14.4 (2–47)	8/13 3.7 ± 5.3 (0–19)	0/15
High-frequency ventilation	4/9 3.4 ± 5.0 (0–12)	0/13	0/15
Binasal continuous-positive-pressure ventilation (CPAP)	27.8 ± 9.5 (14–40)	7.1 ± 5.1 (0–20)	0/15

Data are mean ± standard deviation (range), if not displayed otherwise

With regard to neurological long-term outcome (cerebral palsy, visual and hearing impairments) no significant differences were observed among the groups for any of these. This is also true for physician-diagnosed and parent-reported bronchial asthma (only one child in the No-BPD group was affected by bronchial asthma).

### Lung function

Results of spirometry, airway resistance and diffusion capacity testing are presented in Table 3. Significant differences among groups were detected by ANOVA for forced expiratory volume in 1 s (FEV1% predicted), specific effective airway resistance (SReff% predicted) and the transfer factor of the lung for carbon monoxide (TLCO% predicted). Results of posthoc testing are displayed in Table 4. Although FEV1 was significantly lower in BPD compared with CONTROL and SReff was higher in BPD compared to CONTROL and No-BPD, average values of the BPD group were still within the limits of the normal range. Diffusion capacity was reduced in both preterm groups (BPD and No-BPD). However, only BPD showed a mild average impairment with regard to normal values. Exhaled NO was within normal values in all three groups [31].

**Table 3:** Lung function parameters

parameter	BPD	No-BPD	CONTROL	*p-*value
FEV1 (%pred)	83 ± 22	97 ± 11	105 ± 8	**0.002**
FVC (%pred)	95 ± 17	101 ± 13	106 ± 7	0.09
RV%TLC (%pred)	122 ± 52	109 ± 32	109 ± 28	0.664
SReff (%pred)	204 ± 98	138 ± 39	120 ± 43	**0.006**
TLCO (%pred)	75 ± 16	85 ± 8	92 ± 11	**0.001**
eNO (ppb)	9.7 ± 6.4	14.5 ± 6 .4	5.4 ± 5.3	**0.002**

Data are mean ± standard deviation

Significant *p*-values are marked in boldface

**Table 4:** Posthoc testing results for significant differences in lung function testing

		*p*-value
FEV1 (%pred)	CONTROL vs No-BPD	0.349
	CONTROL vs BPD	**0.001**
	No-BPD vs BPD	0.073
SReff (%pred)	CONTROL vs No-BP	1.0
	CONTROL vs BPD	**0.006**
	No-BPD vs BPD	**0.044**
TLCO (%pred)	CONTROL vs No-BPD	**0.007**
	CONTROL vs BPD	**< 0.001**
	No-BPD vs BPD	0.121

Results are *p*-values of posthoc Bonferroni-testing after comparing groups using ANOVA

Significant *p*-values are marked in boldface

### Exercise capacity

Results of exercise testing are presented in Table 5. All participants reached a maximal effort based on predefined criteria (see above). Maximal work rate (Wpeak) expressed in % predicted did not differ between the groups. However, the groups differed significantly in peak oxygen uptake (ml/min), also when expressed in % predicted. Posthoc testing showed a significant difference between CONTROL and BPD (*p* < 0.05), not between the other groups. In a general linear model, peak oxygen uptake was explained by height (*p* < 0.001, eta squared 0.674) and BPD status (*p* < 0.05, eta sqared 0.136), not by prematurity (*p* = 0.906, eta squared 0). BPD showed a significantly lower tidal volume at peak exercise while achieving the same minute ventilation in comparison to the other groups (see Table 5).

**Table 5:** Results of exercise testing

parameter	BPD	No-BPD	CONTROL	*p*-value
VO2peak (ml/min)	1292 ± 343	1649 ± 388	1508 ± 243	0.050
VO2peak (%pred)	83 ± 11	91 ± 8	94 ± 9	**0.024**
Wpeak (% pred)	97 ± 18	112 ±	112 ± 15	0.088
Peak heart rate (/min)	196 ± 8	197 ± 7	197 ± 7	0.79
Peak SpO2, (%)	97.8 ± 2	98.1 ± 3	99.0 ± 1	0.20
RERpeak	1.05 ± 0	1.13 ± 0	1.08 ± 0	0.43
Vtpeak (ml)	782 ± 199	1108 ± 2829	980 ± 313	**0.036**
VEpeak (l/min)	46 ± 12	58 ± 17	52 ± 9	0.21

Data are mean ± standard deviation

Significant *p*-values are marked in boldface

### Physical activity

**Fig. 2:**
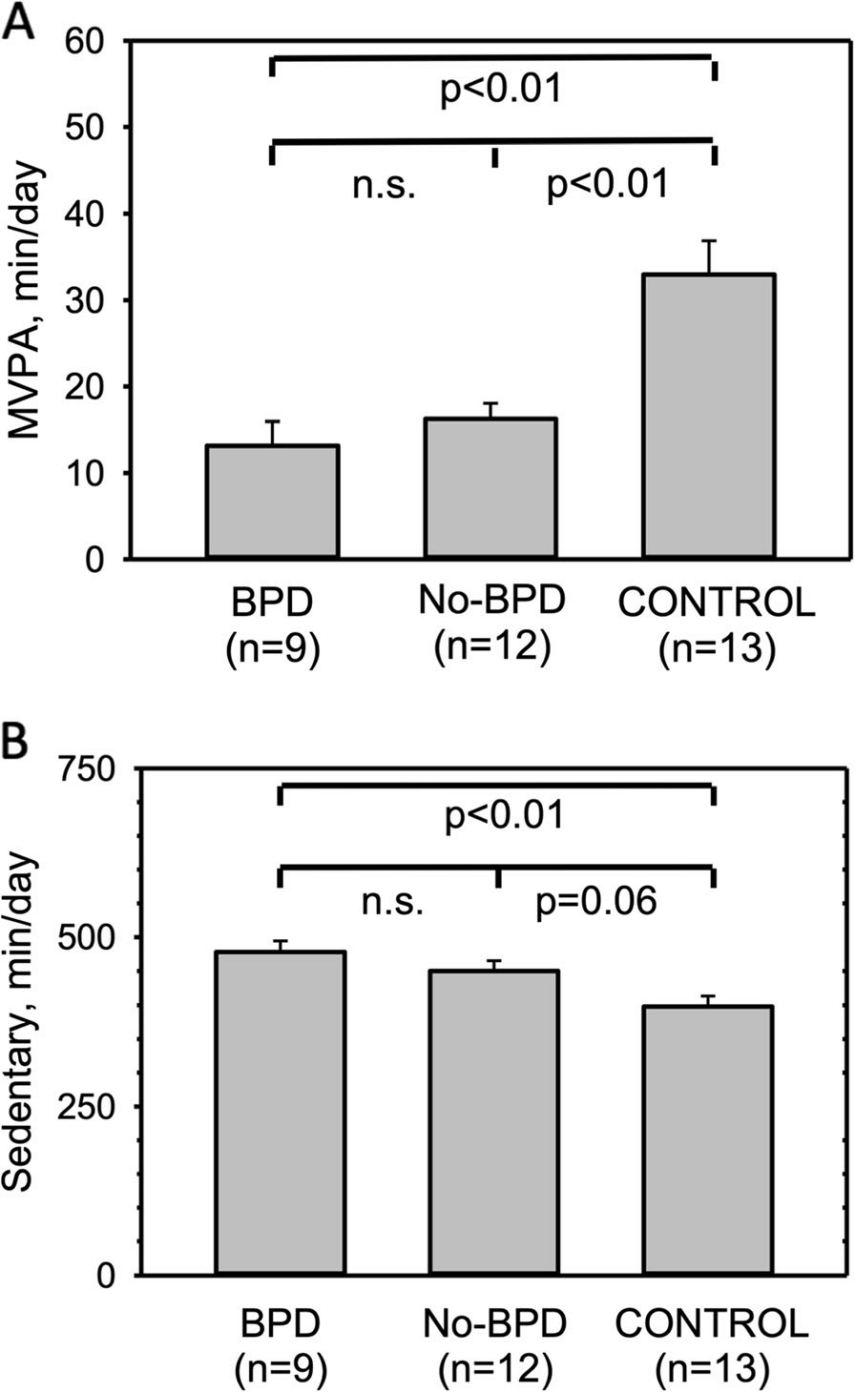
Time spent in two physical activity levels per day as determined from accelerometry in BPD, No-BPD and CONTROL, **a**) Moderate-to-vigorous physical activity, **b**) Sedentary behaviour, Columns represent minutes spent in the respective activity level per day. Data are means plus standard errors. ANOVA showed significant differences among groups for both activity levels (moderate-to-vigorous activity *p* < 0.001; sedentary behaviour *p* = 0.004). Between group differences displayed are based on posthoc test analyses

Results of accelerometry are presented in Fig. 2a and b. The CONTROL group spent significantly more time in MVPA than both preterm groups (Fig. 2a). No difference was observed between BPD and No-BPD. The amount of time spent in sedentary behaviour was significantly elevated in children born preterm (Fig. 2b). Again, there was no difference between BPD and No-BPD.

A general linear model for sedentary behaviour showed that although height had a significant impact on the model (*p* < 0.05, eta squared 0.141), prematurity further contributed significantly to explaining variance (*p* < 0.05, eta squared 0.123), whereas BPD status did not. When replacing height by BMI, only prematurity showed a significant influence on the model (*p* < 0.05, eta squared 0.170).

Likewise, in a general linear model for MVPA only prematurity contributed to explaining variance (*p* < 0.001, eta squared 0.315) while height and BPD status did not.

## Discussion

The major finding of this study was that BPD but not prematurity per se was associated with reduced exercise capacity. However, prematurity regardless of BPD was related to less engagement in physical activity and more time spent in sedentary behaviour at school-age.

In our cohort, BPD but not prematurity was linked to lower exercise capacity and decrements in lung function. A review analysing 22 studies on exercise capacity concluded that children born preterm have lower peak oxygen uptake and that those with BDP show the lowest values [32]. However, differences to term-born controls were rather small and the clinical relevance of this finding remains unclear. Still, reduced peak oxygen uptake may indicate constraints of gas exchange during exercise and suggests that preterm-born children, especially those with BPD, need their ventilatory reserve to achieve equal or nearly equal peak oxygen uptake [32, 33]. This is in line with our data, where the BPD group showed lower peak oxygen uptake and at the same time lower peak tidal volume, possibly reflecting altered breathing mechanisms. There is ample evidence that children born preterm show decrements in lung function (reduced FEV1, FVC, TLCO) [17, 34] and those with BPD are affected even more [16, 17, 35, 36], which may result in reduced exercise capacity [37]. We also observed an impairment in diffusion capacity in the BPD and the No-BPD groups. However, a mild impairment of diffusion capacity did not have any effect on exercise capacity in normoxia and even hypoxia in adults born extremely preterm [15]. Nevertheless, we cannot exclude that the lower diffusion capacity which was most evident in the BPD group contributed to the reduced exercise capacity.

In our cohort, prematurity was related to significantly less moderate-to-vigorous physical activity (MVPA). One of the few reports on physical activity of people born preterm found a significantly lower leisure time activity in otherwise unimpaired adults born preterm and healthy adults [18]. In contrast, other studies reported no difference between preterm and term-born children in physical activity behaviour assessed by questionnaires [38] or accelerometry [3, 20]. In one of the mentioned accelerometry studies, epoch time had been set to 60 s [3], for the other study epoch time is not reported [20]; therefore, short activity bouts typical for children might have been missed. In the Epicure study [3] which used the same MVPA cut-offs as we did, both groups, preterm and term-born children spent very little time in MVPA (9 vs 11 min per day). As the term-born group spent far less time in MVPA compared to other studies on healthy individuals, it may be hypothesized that the comparable MVPA between preterm and term-born children in the Epicure study was due to an unusually poor MVPA in the term-born children rather than a “normal” unimpaired MVPA in the preterm group. Another study with the same MVPA cut-offs reported 25 and 19 min (boys and girls) spent in MVPA per day in children born 25–32 weeks’ gestation compared to 27 and 16 min (boys and girls) in term-born children [20]. Compared to this study, children born preterm of our cohort spent less time in MVPA with an average of 15 min, however, term-born controls were more active as they spent 33 min in MVPA. Spending about half an hour in MVPA per day is in line with activity reported for healthy children [39]. Due to the small sample size we did not perform a gender-specific analysis. However, since there was no difference in gender distribution among groups, the effects observed in this study cannot be attributed to a gender selection bias.

Few studies assessed sedentary behaviour, although it has been depicted as a further independent risk factor for obesity and its sequelae [40]. Since prematurity itself is associated with a risk to acquire high blood pressure, lower bone mineral density and impaired glucose tolerance [41–43], sedentary behaviour may further increase these risks. On the other hand, regular physical exercise has been shown to reduce cardiovascular risk factors, especially in adults with low birthweight [44]. To our knowledge, only one study previously assessed inactivity in children born < 32 weeks’ gestation [45] and reported 14 min/day of additional sedentary behaviour compared to term-born children. However, this difference was not significant. The present study is in line with these findings and is the first to describe a significant difference between term-born children and preterm-born children in sedentary behaviour.

Reduced engagement in physical activity of children born preterm might be of multifactorial origin. Altered breathing mechanisms with an earlier sensation of dyspnoea [5, 32, 46, 47] may lead to refrain from strenuous exercise. Further factors have been identified to affect activity behaviour. Sex, health problems, motor competence and hyperactivity influenced activity behaviour in a cohort of 12- to 20-year-olds born preterm [48]. Adults born at extremely or very low birth weight tend to have less physical self-confidence and a poorer physical coordination [19, 49, 50]. Besides poorer strength, flexibility and motor coordination in seemingly unimpaired adolescents born with extremely low birthweight [19], clumsiness may further lead to lower physical self-confidence and consequently to refraining from regular physical activity. One recent study showed that developmental coordination disorder is much more common than thought and is often underreported by parents [51]. Whether parental overprotection may also lead to less engagement in physical activity needs yet to be clarified.

The strength of this study is that objectively measured physical activity data was generated through accelerometry with 15 s epochs to catch sporadic, short bouts of physical activity typical for children’s play. Most studies used questionnaires to assess activity behaviour; these, however, tend to overestimate activity while at the same time recalling short, intense bouts of activity is difficult for children [52, 53]. Recall bias therefore impairs valid activity data from questionnaires [20, 54].

### Limitations

Limitations of this study are the small sample size, a possible selection bias and the choice of cut-offs for physical activity assessment. Of the invited 36 children with moderate to severe BPD, consent became available for 10 children while no consent was given for 15 children. It may, thus, be speculated that only those with a certain interest in physical activity took part and that these may be the fitter and more active children of this cohort. However, this hypothesis would imply that the real impairments in BPD in exercise capacity and physical activity are even larger than observed in our study.

Activity of all participants was measured using the same cut-off for MVPA. The choice of cut-offs and whether cut-offs should be age-, gender-, height- or weight-specific is constantly under discussion. Since in our cohort, groups did not show significant differences in height, weight and age, the same cut-offs for all participants were chosen, which also enabled us to compare our data to existing research. Further, the accelerometer is worn on the hip which may impair the recording of certain activities. Especially cycling, a popular activity in children, is underestimated with this kind of measurement. As this is similar for all participants, though, we do not believe that this aspect relevantly distorts our results.

Since this is a single centre study focussing on the new BPD era, we have little bias regarding treatment during the neonatal period. Further, only children with moderate and severe BPD were included to clearly distinguish between children born premature with and without BPD.

## Conclusions

According to our results, children with BPD show impaired exercise tolerance, probably due to constraints in lung function. Prematurity itself seems to predispose for reduced engagement in physical activity and a preference for sedentary behaviour. Further research is needed to determine the mechanisms of this behaviour and to analyse whether physical activity intervention programs are feasible and effective to countervail reduced physical activity and increased sedentary behaviour and their negative effects.

## Data Availability

The datasets used and analysed in this study are available from the corresponding author upon reasonable request.

## Abbreviations

BPD: bronchopulmonary dysplasia / children born preterm with moderate or severe bronchopulmonary dysplasia
CONTROL: term-born children
eNO: exhaled nitric oxide
FEV1: forced expiratory capacity in one second
FVC: forced vital capacity
MET: metabolic equivalent of task
MVPA: moderate to vigorous physical activity
No-BPD: children born preterm without bronchopulmonary dysplasia
RER: respiratory exchange ratio
RV/TLC: residual volume to total lung capacity ratio
SReff: specific effective airway resistance
TLCOC: lung diffusion capacity for carbon monoxide
VE: ventilated volume in one minute
VO2peak: peak oxygen uptake
Vt: tidal volume
Wmax: peak work load in Watt
